# Functional Roles of the IgM Fc Receptor in the Immune System

**DOI:** 10.3389/fimmu.2019.00945

**Published:** 2019-05-03

**Authors:** Hiromi Kubagawa, Kazuhito Honjo, Naganari Ohkura, Shimon Sakaguchi, Andreas Radbruch, Fritz Melchers, Peter K. Jani

**Affiliations:** ^1^Deutsches Rheuma-Forschungszentrum, Berlin, Germany; ^2^Department of Medicine, School of Medicine, University of Alabama at Birmingham, Birmingham, AL, United States; ^3^Immunology Frontier Research Center, Osaka University, Osaka, Japan

**Keywords:** FcμR, autoantibody, natural IgM, tolerance, Mott cell, epigenetics

## Abstract

It is now evident from studies of mice unable to secrete IgM that both non-immune “natural” and antigen-induced “immune” IgM are important for protection against pathogens and for regulation of immune responses to self-antigens. Since identification of its Fc receptor (FcμR) by a functional cloning strategy in 2009, the roles of FcμR in these IgM effector functions have begun to be explored. Unlike Fc receptors for switched Ig isotypes (e.g., FcγRs, FcεRs, FcαR, Fcα/μR, pIgR, FcRn), FcμR is selectively expressed by lymphocytes: B, T, and NK cells in humans and only B cells in mice. FcμR may have dual signaling ability: one through a potential as yet unidentified adaptor protein non-covalently associating with the FcμR ligand-binding chain via a His in transmembrane segment and the other through its own Tyr and Ser residues in the cytoplasmic tail. FcμR binds pentameric and hexameric IgM with a high avidity of ~10 nM in solution, but more efficiently binds IgM when it is attached to a membrane component via its Fab region on the same cell surface (*cis* engagement). Four different laboratories have generated *Fcmr-*ablated mice and eight different groups of investigators have examined the resultant phenotypes. There have been some clear discrepancies reported that appear to be due to factors including differences in the exons of *Fcmr* that were targeted to generate the knockouts. One common feature among these different mutant mice, however, is their propensity to produce autoantibodies of both IgM and IgG isotypes. In this review, we briefly describe recent findings concerning the functions of FcμR in both mice and humans and propose a model for how FcμR plays a regulatory role in B cell tolerance.

## Introduction

Two forms of IgM exist that differ in the carboxyl terminus of the heavy chain (HC). Alternative splicing with a transmembrane exon (μm) generates monomeric membrane-bound IgM as a B cell receptor (BCR) for antigen and with a secretory exon (μs) polymeric IgM secreted by plasma cell as a component of humoral immunity. The secreted form of IgM consists mainly of J chain-containing pentamers. The existence of J chain-deficient hexamers has also been reported albeit at an unknown concentration. To determine the role of secreted IgM in immune responses, two different groups have independently disrupted the exon encoding the μs (μ*s* KO) (1, 2). Such mutant mice normally express IgM and other Ig isotypes on the surface of B cells and secrete all Ig isotypes except for IgM. These mutant mice are unable to control infections, because of inefficient induction of a protective IgG antibody response (3–5). Paradoxically, the autoimmune pathology associated with IgG autoantibody is more severe in μ*s* KO mice than in the control mice, possibly because of impaired clearance of autoantigen-containing apoptotic cells (6, 7). Yet, no studies have directly demonstrated such deficiency in removal of self-antigens. Thus, both natural and immune IgM are important for protection against pathogens as well as in regulation of immune responses to self-antigens (8).

A variety of secreted and cell surface proteins is involved in binding the Fc portion of antibody, thereby participating in its effector function, e.g., complement and various types of Fc receptors (FcRs). Classical FcRs for switched Ig isotypes (i.e., FcγRs, FcεRI, FcαR), the receptor for polymeric IgA and IgM (pIgR), the low affinity FcεRII/CD23, and the FcR for neonatal IgG (FcRn) have thus far extensively been characterized at both genetic and protein levels (9–17) (see also other articles in this issue), and much of the knowledge gained has now been translated to clinical practice (18, 19). On the other hand, the role of the IgM FcR (FcμR) as an effector molecule for IgM antibody, the first Ig isotype appearing during phylogeny, ontogeny and immune responses, has just begun to be explored, since the *FCMR* was identified in 2009 (20). Several FcμR review articles have recently been published elsewhere (21–25). Here we briefly reiterate the biochemical structure of the FcμR and its functional roles in the development of B cell subsets and plasma cells, describe the potential molecular bases for certain discrepancies observed among different *Fcmr* KO mice, and introduce our theoretical model for how FcμR is involved in B cell tolerance.

## Unique Properties of FcμR

### Dual Signaling Ability

**Figure 1:**
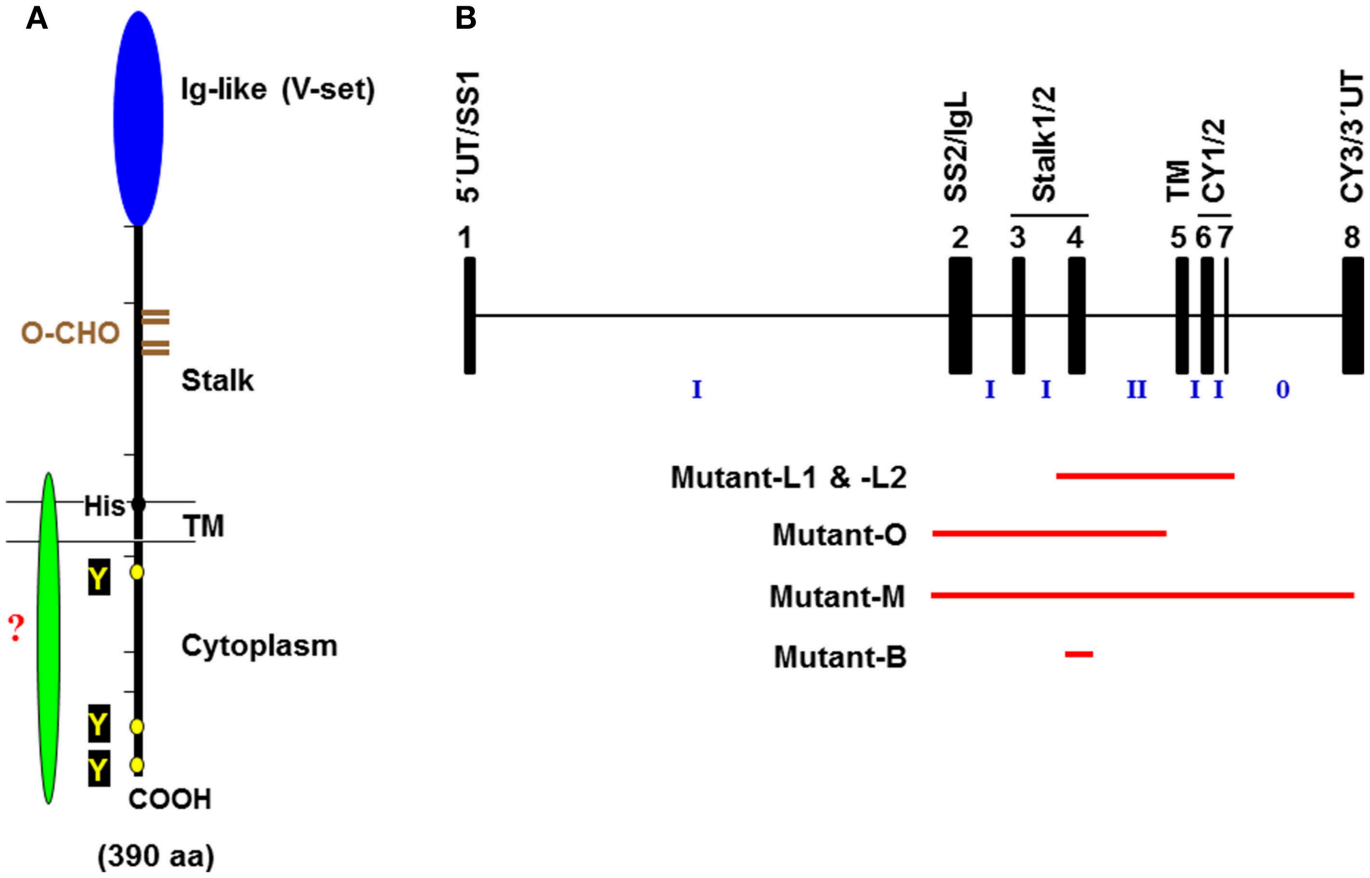
Schematic representation of the FcμR. **(A)** Predicted FcμR protein structure. The human FcμR cDNA encodes a type I transmembrane protein of 390 aa with a peptide core of ~41 kD that consists of a signal peptide (not shown), an Ig-like domain (V-set), remaining extracellular (stalk), transmembrane (TM; between two lines) and cytoplasmic region. Black and brown hatch marks indicate exon boundaries in the *FCMR* gene and O-glycosylation sites, respectively. Small black and yellow circles indicate a TM charged His residue and conserved Tyr residues, respectively. A green fusiform indicates a hypothetical adaptor protein non-covalently associating with the FcμR ligand-binding chain via the His residue. **(B)** Schematic representation of targeted exons in *Fcmr-*ablated mice. The exon (black closed boxes) organization of *Fcmr* is drawn along with intron phases (“phase 0” indicates between the codons; “phase I” between the first and second nucleotide of a codon; “phase II” between the second and third nucleotide). Exons encoding particular regions of the receptor are denoted as follows: the 5′ untranslated (5′UT), the signal peptide (SS1 and 2), the Ig-like domain (IgL), the uncharacterized extracellular (Stalk 1 and 2), the transmembrane (TM), the cytoplasmic (CY1-3), and the 3′ untranslated (3′UT) regions. Red lines indicate the exons targeted in each *Fcmr* knockout mouse strain (see text for details).

*FCMR* is a single copy gene located on chromosome 1q32.2 adjacent to two other IgM-binding receptors *PIGR* and *FCAMR* (FcR for IgA and IgM) (20). The predicted human FcμR is a type I glycoprotein of 390 amino acids (aa) with a peptide core of ~41 kD, which consists of a signal peptide, a V-set Ig-like domain responsible for Fcμ binding, an additional extracellular region with unknown domain structure (termed the stalk region), a transmembrane (TM) segment containing a charged His residue (H^**253**^) and a relatively long cytoplasmic (CY) tail of 118 aa containing conserved, three Tyr and five Ser residues (see Figure 1A). Among these Tyr residues, the carboxyl terminal Y^**385**^ matches the Ig tail Tyr motif (DYxN; x indicates any aa) seen in IgG and IgE (26), but the other two do not correspond to any known Tyr-based signaling motifs, ITAM, ITIM or switch. Two carboxyl terminal Y^**366**^ and Y^**385**^ are involved in receptor-mediated endocytosis (27, 28) and the membrane proximal Y^**315**^ is predominantly involved in the FcμR-mediated protection from IgM anti-Fas monoclonal antibody (mAb)-induced apoptosis (28) (see below). An important role of the H^**253**^ residue in anchoring the receptor in the plasma membrane became evident when the fate of IgM bound to FcμR in cells stably expressing the wild type (WT) or H253F mutant form of receptor was examined by immunofluorescence microscopy; the mutant showed enhanced cap formation even at 4°C. IgM ligand-binding activity was found significantly increased in an FcμR mutant with a deletion of most of the CY tail compared to the WT receptor, despite comparable surface levels as determined by receptor-specific mAbs. Based on our preliminary data, this enhancement appears to result from the formation of an oligomeric FcμR as a consequence of its presumably mobile nature within the plasma membrane. This is different from our speculated inside-out regulation of FcμR ligand binding by its CY tail as seen in integrins. Ligation of FcμR with preformed soluble IgM immune complexes induced phosphorylation of both Tyr and Ser residues (20). Intriguingly, the phosphorylated FcμR migrated faster on SDS-PAGE than the unphosphorylated form, unlike most proteins that run slower when phosphorylated. Preliminary data with an epitope-tagged FcμR suggest that there could be cleavage of the CY tail of FcμR, but the precise molecular mechanisms for this cleavage and the functional role of the resultant FcμR stub still need to be elucidated. Collectively, these features of human FcμR suggest a dual signaling ability of FcμR: one via a potential as yet unidentified adaptor protein non-covalently associating with the FcμR via the H^**253**^ residue and the other from its own Tyr and Ser residues in the CY tail.

While mouse ortholog with 422 aa has relatively low homology (~54%) with human FcμR, the overall structural characteristics (a single Ig-like domain, a His residue in TM segment, and a long CY tail containing three Tyr and five Ser residues) are conserved. However, the analysis of its biochemical nature including the ligand binding is limited (22, 29).

### Lymphocyte-Restricted Distribution

Given the fact that IgM is the first Ig isotype to appear during phylogeny, ontogeny and immune responses, we initially thought that FcμR would have a broad cellular distribution, thereby serving as a first line of defense against pathogens. On the contrary, FcμR was found to be expressed by lymphocytes only: both B and T cells and, to a lesser extent, NK cells in humans, and only B cells in mice (20, 29–32). Unlike the phylogenetically broad distribution of IgM from jawed vertebrates onward (i.e., cartilaginous fish), computational analysis of existing genomic sequence databases unexpectedly reveals that FcμR appears probably in early reptiles and is found in all three major living (extant) groups of mammals (i.e., egg laying, marsupial and placental mammals) (33). FcμR is the only FcR constitutively expressed on human T cells, which are otherwise generally negative for FcRs, and for B cells, FcμR is the only IgM-binding FcR expressed. [In this regard, another IgM-binding receptor, Fcα/μR, was initially reported to be expressed by B cells, but subsequent analyses revealed that the major cell type expressing Fcα/μR in immune system is a follicular dendritic cell in both humans and mice (34).] During B-lineage differentiation, the cell surface expression of FcμR was detectable from pre-B/B transitional stage to plasmablasts, except for a transient down-modulation during germinal center reactions in both humans and mice (20, 29, 30, 32, 35). Collectively, the restriction of FcμR expression to adaptive immune cells is thus remarkable, because FcRs for switched Ig isotypes are expressed by various hematopoietic cells including myeloid cells as central mediators coupling innate and adaptive immune responses. Lymphocyte-specific FcμR may thus have a distinct function from myeloid cell FcRs.

### *Cis* Engagement

**Figure 2:**
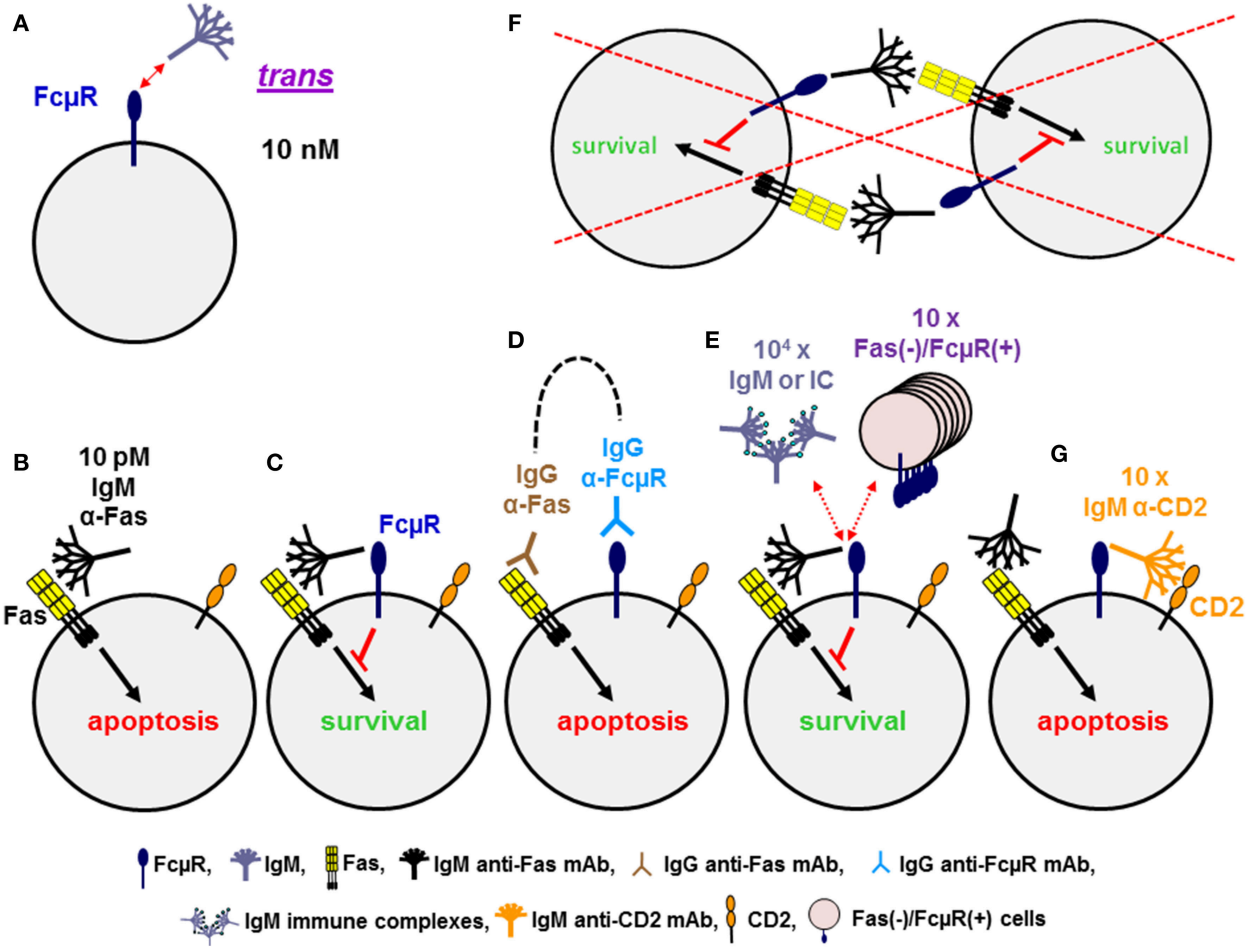
Dominant *cis*, rather than *trans*, interaction of FcμR. **(A)** FcμR positive cells bind IgM pentamers in solution in *trans* with high avidity of ~10 nM. **(B–G)** Ligation of Fas death receptor trimer with agonistic IgM anti-Fas mAb induces apoptosis in WT Jurkat cells **(B)**, but not in FcμR-positive Jurkat cells **(C)**. Co-ligation of Fas and FcμR with the corresponding IgG mAb plus a common secondary reagent (dotted line) has no inhibitory effects on the IgG Fas mAb-induced apoptosis **(D)**. FcμR-mediated protection from IgM Fas mAb-induced apoptosis is not blocked by addition of 10^4^ molar excess of IgM or its soluble immune complexes or 10-fold excess of Fas(–)/FcμR(+) cells, suggesting an efficient *cis* interaction of IgM Fas mAb and FcμR on the same cell surface **(E)**, but not a *trans* interaction between neighboring cells **(F)**. Addition of tenfold excess of IgM mAb reactive with CD2 on Jurkat cells can efficiently block the interaction of IgM Fas mAb and FcμR, resulting in apoptosis **(G)**.

Cell surface FcμR in humans is a sialoglycoprotein of ~60 kD and one third of the relative molecular mass of the mature FcμR is thus made up of O-linked glycans. It exclusively binds the Fc portion of pentameric and hexameric IgM with strikingly high avidity of ~10 nM as determined by Scatchard plot analysis with the assumption of a 1:1 stoichiometry of FcμR to IgM (20, 25) (Figure 2A). Much higher concentrations (>100-fold) are required for binding of monomeric IgM to FcμR-bearing cells, indicating the importance of IgM conformation. This in turn suggests that serum IgM, at its serum concentration of ~1 μM, constitutively binds to FcμR on the surface of lymphocytes. In addition to the high avidity for IgM in solution, a unique ligand-binding property of FcμR was observed when IgM mAbs to lymphocyte surface proteins were used as a ligand. When Fas death receptor is ligated with 10 pM agonistic IgM anti-Fas mAb, apoptosis-prone Jurkat cells undergo robust apoptosis within 1 day, but Jurkat cells stably expressing FcμR do not (Figures 2B,C). This finding is thus consistent with previously reported anti-apoptotic activity of Toso (the original name of FcμR) (36). [In this review we will only use “FcμR” as the name of the receptor, based on a recent nomenclature agreement (37).] However, ligation of Fas with agonistic IgG3 anti-Fas mAb or co-ligation of both Fas and FcμR with the corresponding mouse IgG mAbs plus an appropriate common secondary reagent [e.g., F(ab')_2_ fragments of anti-mouse γ antibody] had no inhibitory effects on the IgG3 Fas mAb-induced apoptosis (Figure 2D). This suggests that FcμR *per se* has no intrinsic activity to inhibit Fas-mediated apoptosis. The anti-apoptotic activity of FcμR depends on usage of the IgM Fas mAb and not on physical proximity of two receptors by artificial co-ligation as observed with ITIM containing receptors such as FcγRIIb and paired Ig-like receptor B (38, 39).

To determine whether the interaction of the Fc portion of IgM Fas mAb with FcμR occurs in *cis* or *trans*, a 10-fold excessive of Fas(–)/FcμR(+) cells as a potential competitive source of FcμR(+) cells was added into the assay but no inhibition of FcμR-mediated protection of Jurkat cells was observed (Figure 2E). This suggests that the interaction of the Fc portion of IgM Fas mAb with FcμR occurs in *cis* on the same surface of Jurkat cells (Figure 2E), but not in *trans* between neighboring cells (Figure 2F). Addition of >10^4^ molar excess of IgM or its soluble immune complexes was required for partial, but significant blockade of such a *cis* interaction (Figure 2E), suggesting that the soluble IgM immune complexes are not potent competitors in the FcμR-mediated protection from IgM Fas mAb-induced apoptosis. However, when IgM mAb reactive with other surface proteins expressed on Jurkat cells (e.g., CD2 or TCR) was used as a potential competitor, a 10-fold excess of IgM anti-CD2 or anti-TCR mAb was sufficient to block the *cis* interaction, thereby permitting FcμR(+) cells to undergo apoptosis (Figure 2G) (28). Similar results with agonistic IgM vs. IgG3 Fas mAb were observed with Epstein Barr virus-transformed B cell lines simultaneously expressing endogenous FcμR and Fas on the cell surface (20). Furthermore, when BCR and FcμR on blood B cells were co-ligated with a mitogenic IgM anti-κ mAb in the presence of IgG2b anti-FcμR mAbs with blocking or non-blocking activity for IgM-ligand binding, Ca^**2+**^ mobilization was the same in the absence or presence of FcμR non-blocking mAb. By contrast, the FcμR blocking mAb significantly diminished Ca^**2+**^ mobilization by blood B cells, suggesting that FcμR provides stimulatory signals upon BCR cross-linkage with IgM mAbs (28). Collectively, these findings of human FcμR indicate that although FcμR binds soluble IgM pentamers and hexamers at a high avidity of ~10 nM, FcμR binds more efficiently to the Fc portion of IgM when it is attached to a membrane component via its Fab region on the same cell surface. FcμR expressed on lymphocytes may thus have a potential to modulate the function of target antigens or receptors when they are recognized by natural or immune IgM through its *cis* engagement.

In summary, FcμR: (i) is expressed by lymphocytes: B, T and NK cells in humans and only B cells in mice, suggesting that FcμR may have a distinct function compared to other FcRs, which are mainly expressed by myeloid cells, and potential species differences; (ii) may have dual signaling ability: one from a potential adaptor protein that non-covalently associates with FcμR ligand-binding chain via H^**253**^, and the other from its own Tyr/Ser residues in the CY tail; and (*iii*) binds more efficiently to the Fc portion of IgM when it is attached to a membrane component via the Fab region on the same cell surface (*cis* engagement), than to the Fc portion of IgM in solution/fluids.

## Variant Results Observed in Different *Fcmr-*Deficient Mice

Despite the initial prediction of embryonic lethality of *Fcmr* ablation (40), there are now five different *Fcmr* KO mice that have been independently generated by four different groups of investigators [Lee et al. (mutant-L1 and -L2), Ohno et al. (mutant-O), Mak et al. (mutant-M), and Baumgarth et al. (mutant-B)]. Eight different groups of investigators have characterized these mutant mice with clear differences in reported phenotypes (29, 32, 35, 41–50) (see Table 1). This is an unusual case in the gene-targeting field. Several discrepancies could be in part due to the following: (i) Investigator's preconception of FcμR or Toso in terms of its cellular distribution (B cells vs. myeloid and T cells) (51, 52) and its function (binding IgM Fc vs. inhibiting Fas- or TNFα-mediated apoptosis) (53, 54). (ii) Differences in embryonic stem cells of C57BL/6 (mutant-L1, -L2, and -B) vs. 129/sv (mutant-O and -M) origin and the extent of the 129 mouse-origin DNA still present around the disrupted *Fcmr* gene after backcrossing onto C57BL/6 background; (iii) Differences in exon targeting strategies [exon 2-4 (mutant-O), 2-8 (mutant-M), 4 (mutant-B) vs. 4-7 (mutant-L1 and -L2) (Figure 1B)], global (mutant-O and -M) vs. conditional deletion (mutant-L1, -L2, and -B), and the *Cd19* heterozygosity in the CD19-Cre-mediated deletion vs. the unmanipulated *Cd19* in global deletion, and the presence (mutant-M) vs. absence (other mutants) of the *Neo* gene in the mouse genome; and/or (iv) other factors, e.g., ages of the mice examined, experimental procedures/conditions, environmental factors including intestinal microbiota, or reagents used.

**Table 1:** Phenotypic comparison of five different *Fcmr*-deficient mice.

***Fcmr* KO created by**	**Lee et al. (mutant-L1)**	**Ohno et al. (mutant-O)**					**Mak et al. (mutant-M)**					**Baumgarth et al. (mutant-B)**	**Lee et al. (mutant-L2)**
**Δ exons; ES cell origin**	**4–7; C57BL/6**	**2–4; 129/Sv**					**2–8; 129/Sv**					**4; C57BL/6**	**4-7; C57BL/6**
***Neo* cond. KO**	**Removed Cond. KO (EIIa-Cre)**	**Removed Global del. (backcrossing with C57BL/6)**					**Not removed Global del. (backcrossing with C57BL/6)**					**Removed cond. KO (CD19-Cre)**	**Removed cond. KO (CD19-, CD4- or CD11c-Cre)**
**References (year)**	(41) (2011)	(29) (2012)	(45) (2014)^*^	(32) (2012)	(47) (2015)	(49) (2018)	(42) (2013)	(43) (2013)	(44) (2014)	(46) (2015)	(48) (2015)	(35) (2017)	(50) (2018)
**Distribution of FcμR+** **cells**	B68 mAb: CD4 T, CD8 T, NKT, B & Leuko. in Sp, LN, blood	MM3 mAb: B cells; immat. B to plasma-blast in BM, Sp, LN	*NR*	4B5 mAb: B cells	4B5 mAb: B cells	MM3 mAb: B cells	mRNA: B, Gr-1+, CD11c+ in Sp	B68 mAb: granulo. & mono. in BM, Sp	*NR*	*NR*	*NR*	4B5 mAb: B cells; TGN in immat. B in BM	B68 mA: B cells
**FcμR levels & function in WT mice**	α-CD3/CD28 Rx of CD8 T: ↑	Sp: FOB > MZB > NFB; B1 = B2 PerC: B1a = B2 > B1b		BM: immat. B >proB/preB Sp: MZB > FOB PP: non-GCB > GCB	α-μ Rx of B: ↑; co-IP of FcμR/IgM BCR; α-μ & α-FcμR Rx: ↑p52,↑BCL-xL		BM: immat. B > pro-B, pre-B; Sp: FOB > MZB > GCB, PC; PerC: B2 > B1a;					BM: mat. B > immat. B > late preB; Sp: MZB > B1 > FOB;	BM: mat. B > immat. B, preB
**Lymphocyte populations in BM**	*NR*	Not changed	CD19+ cells↓	Not changed	*NR*	Not changed	cells↓, proB↓, preB↓, immat. B↓, mat.B →	*NR*	*NR*	*NR*	*NR*	B1, B1a, B1b →	Not changed
**Lymphocyte populations in periphery**	Sp, LN and Blood: B↓, CD8 T → , CD4 T →	Sp: B → , FOB → , MZB↓, B1 ↑; LN: B → , T → ; PerC: T↑	Sp: B↓, B2↓, B1a & 1b → , MZB↓, PC↓	Sp:B → , FOB → , MZB↓, Tr3B↓, IgM^**hi**^IgD^**hi**^↑	*NR*	Sp: MZB↑ & FOB↑ at 3wk; MZB↓ at 9 wk	Sp: cells↓, FOB↓; PerC: B1a↑, B2↓	*NR*	*NR*	*NR*	*NR*	Sp: B1↑, B1a↑, FOB↑, MZB → ; PerC: B1 → , B1a → , B1b → ; splenomegaly (8 mo)	Sp: TrB↑, mat. B → , B1↑, B2 → ,FOB → , MZB↑; PerC: B1a↑, B1b↓
**Effect of** ***Fcmr-*****ablation**	↑Suscept. of act. CD8 T to TNFα-induced apoptosis Resistance to TNF/GalN-induced, iNKT-mediated liver damage	↑Basal serum IgM & IgG3 ↑Nat. autoAb of IgM, IgG3 & IgG2c ↑Ab resp. to TI-2 Ag (subopt. dose) ↓1° IgG1 & ↓2° IgM Ab resp. to TD-Ag	↑Basal serum IgM & IgA at early age ↑Nat. autoAb of IgM & IgG ↑MZB-derived IgM α-Sm/RNP Ab Rapid PC- diff. of MZB cells ↑Mott cell formation	↑Basal serum IgM ↑Nat. autoAb of IgM & IgG ↓Ab resp. to TI & TD Ags ↓Survival after BCR ligation ↓GC formation ↓Memory & PC-diff.	↓Survival of B cells upon α-μ Rx → BCR-mediated endocyto → pIκBα & ↓BCL-xL after α-μ Rx	↑Cell death & turnover of MZB cells ↑IgD and MHC II on MZB cells ↓tonic BCR signaling ↓Ab resp. to TI-1 Ag ↑Suscept. to sepsis	Basal serum ↓IgG1 (3 mo), ↑IgG3 & IgA (6 mo) ↑nat. IgG autoAb Ab resp. to: TI-1↑, TI-2 → , TD 1° IgG↓ & 2° IgG → ↓prolif. & survival after α-μ Rx	→ Myeloid development ↑ROS after fMLP Rx of granulocytes ↓phagocyte *Listeria* ↓TNFα, IL-6 after LPS Rx resistance to endotoxin shock *in vivo* suscept. to *Listeria* inf.	Resistant to MOG-EAE ↓T & Mϕ infiltration in brain → CD4 T function ↓DC act. & maturation ↑Treg diff.	↓Recruit. and act. of iDC in LCMV-liver ↓act. of CD8 T in liver fail to induce autoimmune diabetes	↓IL17A, ↓IL10 & ↑INFγ in Th17-polarizing cells	↑IgM BCR ↑tonic BCR signaling ↑basal serum IgM ↑ASC of IgM & IgG in Sp, BM ↑nat. autoAb of IgM & IgG ↑survival of unstimulated B1 & B2 cells.	↑Suscept. to H1N1 infection in CD19-Cre mice only → Ag-specific CD4 & CD8 T resp. ↓TNFα & INFγ by CD4 & CD8 T ↓B cell survival ↑IL10+ Breg → basal serum IgM & IgG ↑natural autoAb of IgM &IgG

* *Mutant-O mice crossed with Fas-deficient autoimmune prone B6/Ipr strain*.

*α-, anti-; Ab, antibody; act., activation; Ag, antigen; ASC, antibody secreting cell; BCR, B cell receptor; BM, bone marrow, cond., conditional; del., deletion; diff., differentiation; FOB, follicular B cells; GCB, germinal center B; iDC, inflammatory dendritic cell; immat., immature; inf., infection; iNKT, invariant NKT; KO, knockout; LN, lymph node; MZB, marginal zone B; nat., natural; NFB, newly formed B; NR, not reported; PC, plasma cell; PerC, peritoneal cavity; PP, Peyer's patch; prolif., proliferation; ROS, reactive oxygen species; Rx, treatment; recruit., recruitment; Sp., spleen; subopt., suboptimal; suscept., susceptibility; Sm/RNP, Smith antigen/ribonuclear protein; TD, T cell dependent; TGN, trans-Golgi network; TI, T cell independent; Tr(3), transitional (3); WT, wildtype; 1°, primary; 2°, secondary; ↑, ↓ & → , increased or enhanced, decreased or diminished, & comparable to WT control mice*.

Another factor that could contribute to these discrepancies is the relative difficulty in assessing cell surface FcμR in mice by flow cytometry using receptor-specific mAbs, because of its relative low cell surface density as well as its sensitivity to extracellular IgM concentrations, tissue milieu and cellular activation status (20, 29, 35). This vulnerability could result in the discrepancy in reported cellular distribution of FcμR in mice. In fact, using the same receptor-specific rat mAb (B68 clone), FcμR was expressed by: mouse B cells (55), myeloid cells (43) or CD4 T, CD8 T, and B cells (41). This conflicted cellular distribution data about FcμR is a major reason why some investigators created additional Cre/loxP-mediated, cell type-specific *Fcmr* deletion systems (35, 50). In this regard, EIIa-Cre mediated *Fcmr-*deleted mutant-L1 (equivalent to global deletion) showed more TNFα-induced apoptosis of CD3/CD28-activated CD8 T cells than control mice (41). The abnormality was initially considered as an intrinsic T cell defect since this group originally reported that FcμR was expressed by T cells. However, subsequent results from conditional deletion clearly indicated no phenotypic differences between T cell-specific [or dendritic cell (DC)-specific] *Fcmr* deletion and control counterparts. The only differences were seen with B cell-specific *Fcmr* deletion. The authors thus concluded that FcμR on B cells might indirectly affect certain T cell functions (50), although it remains unclear how this would work. In this review, we will focus on the following aspects of B cell-related findings in *Fcmr* KO mice: (i) alterations in B cell subsets, (ii) IgM homeostasis, and (iii) dysregulated humoral immune responses.

### Alteration in B Cell Subsets

The development of B-lineage cells in the bone marrow (BM) was unaffected in most *Fcmr* KO strains (29, 32, 35, 50) except for mutant-M where the numbers of pro-B, pre-B, and immature B cells were significantly diminished as compared to WT controls (42). Since the surface expression of FcμR begins to be detectable at the transitional stage of pre-B to B cells in differentiation, it seems conceivable that FcμR is dispensable in developing B-lineage cells in the BM. However, it is noteworthy that: (i) μ*s* KO mice, which are deficient for secretion of IgM, have significantly altered B cell development from pre-B to the immature B cell transition (42); (ii) this alteration of early B cell development is corrected by administration of natural IgM (56); and (iii) many of the abnormalities observed in *Fcmr* KO mice mirror those seen in μ*s* KO mice. Thus, despite the fact that FcμR is a key sensor of secreted IgM, it remains to be elucidated why, among five *Fcmr* KO mice, only mutant-M has an alteration in development of B cell precursors (42). In this regard, several human pre-B cell lines express FcμR transcripts but not FcμR protein on their cell surface at detectable levels unless stimulated with phorbol myrystate acetate (20, 57), suggesting the existence of post-transcriptional controls of FcμR.

Unlike in the BM, in peripheral lymphoid organs there were variable alterations in B cell subsets observed in these mutant mice but, as a general trend, *Fcmr* ablation was found to more profoundly affect innate-like B cells, B-1, and marginal zone (MZ) B cells, rather than the B-2 or follicular (FO) B cell compartment (see Table 1). Remarkably, an increase in B-1 B cell numbers, particularly in spleen accompanied by elevated levels of autoantibodies of both IgM and IgG isotypes, has been the sole result consistently observed in all five mutant mice. Thus, FcμR plays an important regulatory role in the homeostasis of B-1 B cell development and autoantibody production (see further discussion below).

For MZ B cells, the mutant-O had age-dependent alterations in their cell numbers, i.e., increase in young (3-wk) and marked decrease in old (>9-wk) mice (49). This age-dependent reduction of MZ B cells might result from their rapid differentiation into plasma cells in the absence of FcμR, as evidenced by the markedly elevated IgM autoantibodies to Smith antigen/ribonuclear protein, which are considered to be derived from MZ B cells (45). Alternatively, FcμR-deficient MZ B cells might undergo cell death due to lack of survival signals through FcμR upon BCR cross-linkage (49), as shown by cross-talk downstream of FcμR and BCR signaling via the non-canonical NFκB pathway (47). Notably, the reduction of MZ B cells was also observed with both *Fas*- and *Fcmr*-deficient, autoimmune-prone B6.MRL.*Fas*^lpr/lp*r*^*/Fcmr* ^**−/−**^ mice (45). In mutant-M, unlike mutant-O, there were no changes in the MZ B cell compartment, whereas in both CD19-Cre-mediated deletion mutant-B and -L2, the number of MZ B cells was not reduced (for mutant-B) or enhanced (for mutant-L2) (35, 50). Since the number of MZ B cells in μ*s* KO mice is increased by 3-fold and this increase can be normalized by passive administration of natural and polyclonal, but not monoclonal, IgM, we initially considered that the FcμR and its signals upon IgM-ligand binding might play an important regulatory role in the fate of MZ B cells. This simplistic view, however, may need further consideration based on the above conflicting results.

In addition to the aforementioned changes in cell numbers of B cell subsets, there were some differences in the density of certain cell surface markers (e.g., CD19, CD21, CD23, IgD, IgM) between *Fcmr* KO and WT controls (29, 49, 50). The surface levels of several co-receptors of the BCR complex such as CD21 and CD23 were diminished in certain B cell subsets from mutant-O compared to WT controls (29, 50). This was also the case with CD19 that was also significantly diminished on BM immature B cells, but not on BM recirculating and splenic B cells in mutant-O (our unpublished observation). Notably, the surface IgD levels were higher on splenic MZ B in mutant-O mice than WT controls (49). Indeed, our subsequent analysis of the same mutant mice also revealed higher expression of surface IgD on BM recirculating and splenic MZ B cells, but not on FO B cells, than WT controls (unpublished). The molecular basis for this elevated surface IgD density in mutant-O is unclear, but it has been shown that functionally hypo-responsive anergic B cells are characterized by high levels of IgD BCR and generally turn over rapidly when competing, non-tolerant B cells are present (58, 59).

For surface IgM in mutant-O, IgM staining with fluorochrome-labeled anti-μ mAb, which might include endogenous membrane-bound IgM plus cytophilic IgM bound via FcμR or other potential IgM-binding proteins/receptors, was indistinguishable in these B cell populations including BM immature B cells (29, 49). By contrast, in mutant-B the cell surface expression of IgM BCR was significantly increased as compared to control mice, but this phenotype was only demonstrable 3 days after transferring of *Fcmr-*deficient or control B cells into μ*s* KO mice to avoid the influence of cytophilic IgM (35). The authors implied that this increase in IgM BCR in *Fcmr* KO mutant-B was due to the lack of FcμR-mediated constraints on the IgM BCR (see below), resulting in enhanced tonic BCR signaling, facilitating the spontaneous differentiation of B-1 B cells and the increase in autoantibody production. Stimulated emission-depletion microscopic analysis revealed a strong interaction of FcμR with membrane-bound IgM in the *trans*-Golgi network (TGN) of BM immature B cells, but a weak interaction with the IgM on the plasma membrane in mature B cells, thereby constraining transport of IgM to the plasma membrane. This effect on the exocytotic pathway was proposed to regulate surface expression of IgM and eventually limiting tonic IgM BCR signaling. When we examined the potential interaction of FcμR with IgM BCR on the plasma membrane by fluorescence resonance energy transfer, we also found a very low incidence of such an interaction. By contrast, another group showed the physical interaction of FcμR and IgM BCR on the plasma membrane of mature B cells by confocal microscopy (47) and that tonic BCR signaling was diminished in *Fcmr* KO mutant-O (49). Given the low avidity of FcμR for monomeric IgM in solution, it remains unclear how FcμR could interact with membrane-bound IgM in the TGN of BM immature B cells or on the plasma membrane of mature B cells.

Another remarkable finding related to this issue came from immunofluorescence confocal microscopic analysis: strong staining of intracellular FcμR in a region corresponding to the TGN in murine BM immature B cells (35). The results were in close agreement with the findings of FcμR-mediated endocytosis of IgM by chronic lymphocytic leukemia (CLL) B cells in humans (27). The bulk of the intracellular FcμR protein resided in the TGN and in small vesicles, probably sorting endosomes of CLL cells. While the major function of the TGN is to sort proteins destined for the plasma membrane, endosomal compartment or specialized secretory granules, retrograde transport in the endocytic route to the TGN has been demonstrated for several proteins (60). It is thus worth considering whether DNA- or RNA-containing autoantigens are engulfed into endosomes by IgM BCR on immature B cells in the BM, two thirds of which are known to be autoreactive at least in humans, followed by retrograde transport to the TGN where TLR9 or TLR7 recognizes the respective DNA or RNA/IgM BCR complexes and then FcμR binds the Cμ3/Cμ4 of the resultant oligomerized IgM BCRs in the TGN.

### IgM Homeostasis

The pre-immune serum level of IgM or natural IgM was elevated in most *Fcmr* KO mice (29, 32, 35, 50) except for mutant-M (42) and this elevation correlated with the number of *Fcmr* null mutant alleles (*Fcmr* ^**−/−**^ > *Fcmr* ^**+/−**^ > *Fcmr* ^**+/+**^) (32). The frequency of IgM-secreting cells in spleen and BM was significantly higher and the spot sizes in ELISPOT assays were also bigger in mutant-B than their control counterparts (35). FcμR was not expressed by phagocytic cells in spleen and liver including liver sinusoidal endothelial cells, which are thought to be the primary site of IgM catabolism at least in rat, as determined by both immuno-histological and RT-PCR analyses (29). The half-life of injected IgM was comparable between *Fcmr* KO (mutant-O) and WT mice. Thus, the increase in serum IgM levels in naive *Fcmr* KO mice is the consequence of lack of FcμR-mediated regulation of natural IgM production either at the B cell or plasmablast stage in innate-like B cells (29).

### Dysregulated Humoral Immune Responses

Antibody responses to T cell-independent (TI) and T cell-dependent (TD) antigens were dysregulated in *Fcmr* KO mice as compared to WT controls, although there were some differences among mutant mice that might result from differences in mouse ages, antigen doses and forms, administration routes, kinetics, etc. Generally, mutant mice exhibited enhanced TI type 2 responses (involving multiple BCR cross-linkage) but impaired TD responses, especially at suboptimal doses. Since similar selective enhancement of TI-2 immune responses are also observed in μ*s* KO mice (2) and mice deficient for components of the BCR complex such as CD19 (61) or CD81 (62), FcμR seems to regulate B cell responses to TI-2 and TD antigens by interacting differently with BCR complexes on the plasma membrane.

In summary, there are clear differences in reported phenotypes in five different *Fcmr* KO mice in terms of development of B cell subsets and plasma cells, IgM homeostasis and humoral immune responses. However, the increase in B-1 B cell compartment accompanied by elevated levels of autoantibodies of both IgM and IgG isotypes is the sole result consistently observed with all these mutant mice.

## Epigenetic Findings in the *Fcmr-Il10* Locus in Treg Cells

One of the biggest discrepancies in the field is the cellular distribution of FcμR in mice (B cells vs. non-B cells). While several groups of investigators described the predicted functions of FcμR in non-B cell populations, their actual evidence for the surface expression of FcμR by myeloid, dendritic and T cells was rather weak (41, 43, 51, 52). Most of their functional results came from the comparative analysis in chimeras adoptively transferred by a mixture of *Fcmr* KO and WT BM cells or the direct comparison of cellular function between *Fcmr* KO and WT controls (43, 44, 46, 48). This was the reason why the phrase “functional relevant expression of FcμR” by non-B cells was used (52). Nevertheless, several functional outcomes in non-B cells from some *Fcmr* KO mice could be worthy of consideration because of the clear-cut differences compared to WT controls, even though they might be indirect or bystander effects. For example, mutant-M were resistant to the induction of myelin oligodendrocyte glycoprotein (MOG)-induced autoimmune encephalomyelitis (EAE). The authors initially considered that this resistance was not due to an intrinsic impairment of mutant Th1 and Th17 cell functions (see different observations by another group of investigators below), but rather to the immature and tolerogenic nature of mutant DCs, as characterized by their weak inflammatory responses and increased induction of Treg cells (44). Intriguingly, administration of a recombinant soluble FcμR fusion protein, which consisted of the human FcμR ectodomain and human IgG1 Fc (lacking complement binding activity) (FcμR EC/IgG Fc), into EAE-susceptible WT mice resulted in delaying or ameliorating their disease, depending on the time points of injection. While its mode of action was not discussed, it might be possible that since IgM anti-MOG antibody also participates in the demyelination process in EAE, the soluble FcμR EC/IgG Fc could simply act as a decoy receptor.

By contrast, results from recent single-cell RNA sequencing analysis along with complex algorithmic assessments and its functional annotation indicated that FcμR is one of the four critical regulators of Th17 pathogenicity in MOG-induced EAE (48). [The other three included *Gpr65* (G protein-coupled receptor 65)*, Plzp* (promyelocytic leukemia zinc finger transcriptional repressor of the Th2 master regulator *Gata3*) and *Cd5l* (CD5-like antigen, apoptosis inhibitor expressed by macrophages [AIM], or soluble protein α [Spα]). Astonishingly, CD5L/AIM/Spα is a glycoprotein of ~45 kD secreted by macrophages, supports their survival and was originally identified as an IgM binding protein (63–65). Two out of four regulators identified for Th17-mediated EAE were thus capable of binding to IgM, although CD5L/AIM/Spα was annotated as a regulator of lipid biosynthesis (66).] Th17 cells polarized *ex vivo* by differentiation conditions with TGFß+IL-6 or IL-1ß+IL-23+IL-6 from *Fcmr* KO mutant-M were found to secrete significantly less IL-17A and IL-10 than those from control WT mice (48). Mutant naive CD4 T cells exhibited lower FOXP3 levels during Treg cell differentiation upon TGFß stimulation *in vitro*. The authors considered that FcμR could be a negative regulator in a non-pathologic state but a promoter of pathogenicity (48), although it was difficult to understand its mechanisms. Given our findings that none of the sorted T cells with the phenotype of IL-17^+^, INFγ^+^, or IL-17^+^/IFNγ^+^ expressed FcμR transcripts, as determined by gene array analysis (25), it is hard to imagine how such a minor population of Th17 cells expresses functional FcμR, possibly at low levels, on their surface and plays a major regulatory role in the pathogenesis of EAE.

**Figure 3:**
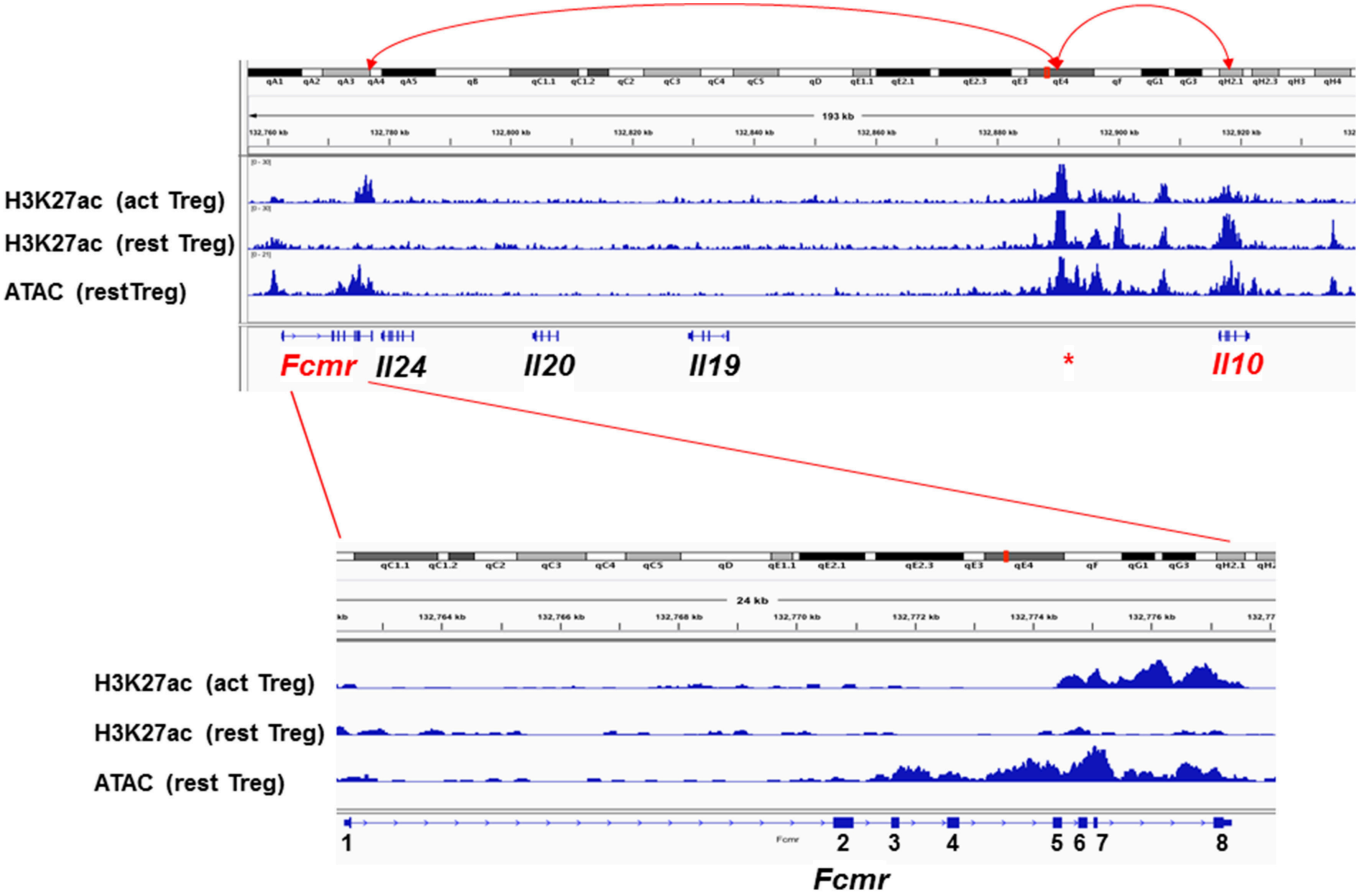
Epigenetic status of the *Fcmr-Il10* locus in Treg cells. Top: Genomic locus (~180 kb) from *Fcmr* to *Il10* is depicted along with the chromosome 1 site designation, distance, marks of the acetylation of histone H3 at lysine 27 (H3K27ac) in activated (act) or resting (rest) Treg cells, marks of the assay for transposonase-accessible chromatin (ATAC) and the exon (square) and intron (line) of indicated genes. Red ^*^ indicates the unique region with high H3K27ac marks in both activated and resting Treg cells and of ATAC at 5′ upstream of *Il10* gene. Red arrow lines indicate potential association with the indicated loci by chromatin loop formation. Bottom: Enlarged illustration of *Fcmr* locus with coding exons numbered. 5′ and 3′ UTR regions are indicated by smaller squares.

To explore the molecular basis for the resistance of *Fcmr* KO mice to EAE as well as for the reduction of IL-10 production by their Th17 cells, a computational epigenetic analysis was performed. Since *Fcmr* and *Il0* genes are ~139 kb apart from each other on chromosome 1, we analyzed the data of the histone post-translational modification by chromatin immunoprecipitation and sequencing and the assay for transposonase-accessible chromatin (ATAC) sequencing available for resting and activated Treg cells at the *Fcmr-Il10* locus (67). These included marks of acetylation of histone H3 at lysine 27 (H3K27ac) as a predictor of enhancer activity (68, 69), albeit not exclusively, and of ATAC as an indication of open chromatin (70). As shown in Figure 3, the H3K27ac marks are selectively observed in three loci, i.e., 3′ site of *Fcmr*, 5′ upstream of *Il10* and *Il10*, in activated Treg cells. The ATAC and H3K27ac marks coincided, suggesting that these loci were in an opened chromatin status, hence transcription factors would be highly accessible to these loci. Remarkably, the H3K27ac marks in the *Fcmr* gene of activated Treg cells were restricted to its 3′ region, i.e., exon 5 (TM) to exon 8 (encoding CY tail and 3′ UTR) and were absent in exon 2 (encoding the Ig-like domain responsible for IgM-ligand binding), consistent with the lack of functional FcμR expression by T cells. This 3′ *Fcmr-*restricted H3K27ac mark was not observed with resting Treg cells, suggesting that the potential enhancer activity of 3′ *Fcmr* in Treg cells was dependent on cell activation. By contrast, the H3K27ac marks in the 5′ upstream of *Il10* were observed irrespective of cell activation. Notably, several regions besides exons in the 3′ *Fcmr* were conserved in 40 other placental mammalian *Fcmr* genes as determined by phastCons (not shown). The above H3K27ac marks were not observed in early B-lineage cells, i.e., pro-B cells of either young or old mice. Collectively, these three loci [3′ site of *Fcmr*, 5′ upstream of *Il10*, and *Il10*] could be involved in enhancing IL-10 expression by Treg cells upon cellular activation potentially through a chromatin loop formation.

While the above epigenetic results of the *Fcmr-Il10* locus were derived from Treg cells, it remained to be elucidated whether a similar scenario was applicable for other cell types including Th17 cells. If so, *Fcmr* KO mutant-M, in which exons 2–8 were targeted, do not have this putative 3' *Fcmr* enhancer element for IL-10 in their genome, and this could account for the reduction of IL-10 production by Th17-polarizing cells (48). For *Il17a*, which is located at ~110 Mb upstream of *Fcmr* on chromosome 1, whether the 3′ *Fcmr* enhancer element is able to form such a long-range interaction with the *Il17a* promoter is an intriguing question. It is also unclear how absence of the 3′ *Fcmr* enhancer element contributes to the resistance to EAE in *Fcmr* KO mutant-M. Nevertheless, given the assumptions that in single-cell RNA sequencing analysis, most identified FcμR transcripts might be derived from its 3′ region and that only the resistance to EAE as the consequence of *Fcmr*-deficiency might be functionally annotated for FcμR, it is thus conceivable and very intriguing that FcμR could be one of the four important regulators of Th17 pathogenicity in EAE, despite the lack of expression of functional FcμR by such T cells (48). Collectively, some of the discrepancies observed in *Fcmr* KO mice could be attributed to differences in the exons disrupted.

In summary, the epigenetic analysis of *Fcmr-Il10* locus reveals that three loci (3′ site of *Fcmr*, 5′ upstream of *Il10*, and *Il10*) may be involved in enhancing IL-10 expression by Treg cells upon cellular activation through chromatin loop formation. The epigenetic alteration selectively at the 3′ site of *Fcmr* may account for the functional abnormalities in non-B cell populations observed in certain *Fcmr* KO mice in conjunction with the exons targeted, even though functional FcμR is not expressed by such non-B cell populations.

## FcμR in Central Deletion of Autoreactive B Cells Developing in Bone Marrow

The common feature among the different *Fcmr* KO mice is the propensity to produce autoantibodies of both IgM and IgG isotypes accompanied by increases in B-1 B cells, indicating an important regulatory role of FcμR in B cell development and central repertoire selection against those B cells expressing autoreactive BCRs. During B cell development in the BM, immature B cells are highly susceptible to deletion by BCR crosslinking. It has been estimated that ~90% of the newly generated BM B cells are deleted before entering the mature B cell compartment (71) and that approximately two thirds of the BM immature B cells in humans are self-reactive (72). During this development, the FcμR expression becomes detectable at the transition from BCR-non-expressing pre-B cells to BCR-expressing immature B cells. In three strains of mutant-O, -B and -L2 (29, 32, 35, 50), however, the sizes of the pro-, pre- and immature B cell compartments showed no alterations, when compared with WT control mice. Only one mutant-M had reduced pro-, pre-, and immature B cell compartments (42). Changes in sizes of BM B-lineage compartment might not become visible in such analyses, because such changes in the number of BCR^**+**^ B cells might occur, as the immature B cells exit the BM. Furthermore, the peripheral compartments of immature and mature B cells may fill by homeostasis to unaltered sizes, though with either non-autoreactive or autoreactive B cells.

**Figure 4:**
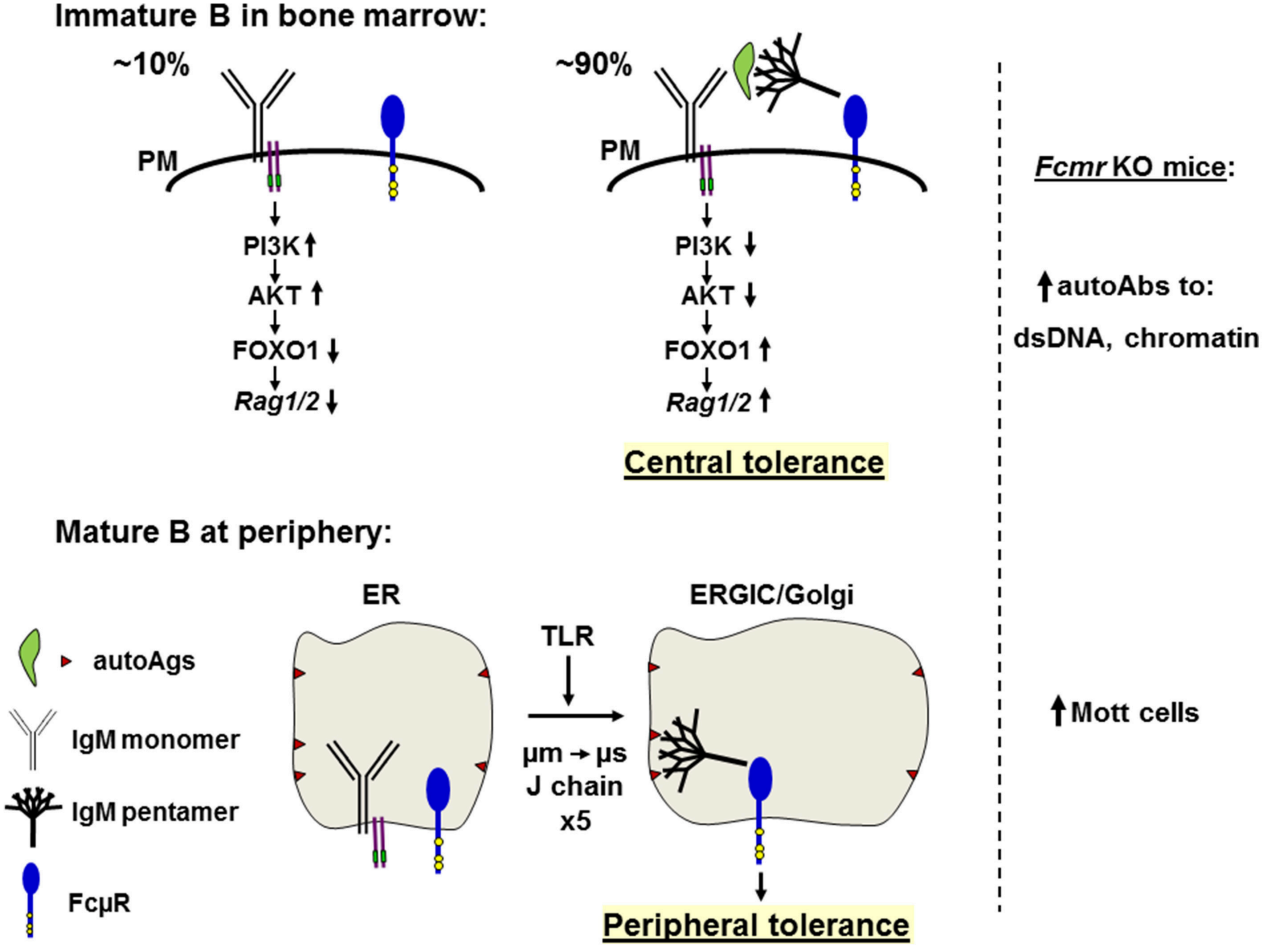
Hypothetical model of the role of FcμR in central deletion and B cell repertoire selection. Top: In the bone marrow, only small populations (~10%) of the newly generated B cells bearing monomeric IgM (Y shape with Igα/Igß, two purple lines carrying green ITAM) on the plasma membrane (PM) are survived by ligand-independent (or tonic) signals through activation of the PI3K-AKT pathway and suppression of the FOXO1-mediated Rag1/2 activity (left). By contrast, ~90% of the newly generated immature B cells have variable binding affinities for autoantigens (green leaf shape) and are subjected to negative selection by receptor editing or apoptosis (right). Autoantigens opsonized with natural pentameric IgM (broom shape) simultaneously bind the corresponding BCR and FcμR (blue tennis racket shape with three conserved Tyr residues in yellow). The resultant cross-linkage of BCR and FcμR on immature autoreactive B cells may inhibit the BCR-mediated PI3K-AKT pathway, resulting in relief of the AKT-mediated suppression of FOXO1 and leading to activation of Rag1/2 and receptor editing, thereby contributing to negative selection or central tolerance. In the absence of FcμR autoantibodies against dsDNA or chromatin are increased. Bottom: In peripheral lymphoid tissues, incompletely edited B cells express IgM BCR with self-reactivity to membrane components (brown triangles) present on ER membranes, but no interaction of monomeric IgM with the corresponding antigens occurs due to its low affinity. When cells receive signals from TLR, a switch from μm to μs exon usage occurs along with the synthesis of J chain during the translocation from ER to the ER Golgi intermediate compartment (ERGIC) or the Golgi and the resultant pentameric IgM is contained inside the vesicles where it binds membrane components via the Fab regions and its Fc portion binds FcμR. This *cis* engagement of self-antigen/secreted IgM/FcμR within the vesicles prevents further development of such autoreactive B cells, thereby contributing to peripheral tolerance. In the absence of FcμR, Mott cells containing intracellular Ig inclusion bodies are increased.

It is noteworthy that μ*s* KO mice, which are deficient for secreted pentameric IgM, the ligand of FcμR, have significantly altered B cell development at the transition from pre-B to immature B cells (42). This alteration of early B cell development, including the inability to centrally delete autoreactive B cells, can be corrected by administration of natural IgM (56). Therefore, ligation of the FcμR by its ligand, pentameric natural, polyclonal IgM *in vivo* contributes to the negative selection of autoreactive B cells. It remains to be elucidated in this experimental setting whether immature B cells in BM are the prime target of this correction. If so, it suggests that the provision of pentameric, natural, polyclonal IgM binding to FcμR on immature B cells allows *cis*-crosslinking of autoreactive BCRs with autoantigen presented by pentameric IgM ligated to FcμR (Figure 4). This crosslinking would be expected to connect signaling from the BCR (e.g., via PI3 kinase) (73) with signaling from FcμR. If FcμR-signaling would downregulate PI3 kinase activity, this could lead to upregulation of FOXO1, which, in turn, could upregulate RAG1/2 expression (74, 75). In this way the immature B cells could continue editing Vl-Jl-rearranged light chain (LC) gene loci (76, 77) to change the autoreactivity of the BCR. Any loss of autoreactivity would abolish *cis-*crosslinking with autoantigen-bound natural IgM/FcμR, thus terminate RAG expression and allow immature B cells to leave the BM.

## FcμR and Mott Cell Formation in the Control of Autoimmunity of B Cells

Another finding is the marked increase in Mott cells in mutant-O, even though it has only been described by our analysis (45). We propose that the FcμR may control autoantibody production by formation of Mott cells in the scenario described below. Mott cells are a variant form of plasma cells containing Ig inclusion bodies (called Russell bodies) that accumulate in dilated rough endoplasmic reticulum (ER). Mott cells are rarely observed in normal lymphoid tissues, but are found in various pathological conditions, such as Ig-associated neoplasms, chronic inflammatory diseases and autoimmune disorders (78–81). Several mechanisms for formation of Ig inclusions or for the defect in Ig secretion have been suggested, including (i) structural alteration of Ig HCs preventing their appropriate processing, (ii) impairment of Ig LCs in preventing Ig HC aggregation, and (iii) inability to degrade or to export Ig, leading to its aggregation. However, the most relevant mechanism associated with mutant-O seems to be that the Ig becomes stuck in the exocytotic pathway due to its autoreactivity with intracellular membrane components. Several precedents support this idea. (i) Two clonally unrelated IgM Mott cell hybridomas utilize germline Ig variable gene segments and have no obvious structural defects, suggesting their B-1 B cell origin (79). (ii) Ig inclusions are not generated when the Mott Ig μ HC or κ LC is by itself or is associated with a heterologous κ LC or μ HC, respectively. The inclusion body formation is only reconstituted when Mott Vh and Mott Vκ genes are expressed with an IgM, but not IgG1, constant region, suggesting that both specificity and isotype are critical for Mott cell formation. (iii) LPS or IL-5 stimulation of sorted B-1 B cells from autoimmune mice (NZB/W F_1_) generates Mott cells *ex vivo* at a frequency of ~50 times higher than conventional B-2 B cells (81). (iv) In studies of autoantibody transgenic mice, incompletely edited B cells express multi-reactive IgM that accumulates in the Golgi and is released or detached from the membrane as insoluble amyloid-like immune complexes termed spherons reaching up to ~2 μm in diameter (82, 83).

Given these precedents and the preferential *cis* engagement of FcμR, the following scenario would account for the high incidence of Mott cells in the absence of FcμR. Incompletely edited B cells migrate into peripheral lymphoid tissues and express membrane-bound IgM with self-reactivity to intracellular membrane components (e.g., glycans). The interaction of the monomeric IgM with self-antigens in the ER must be of low affinity. However, when cells receive certain signals such as from TLR4 to facilitate a switch in the usage of μm to μs exon along with J-chain synthesis during transition to the ER-Golgi intermediate compartment (ERGIC) or the Golgi, the resultant pentameric IgM is contained inside the ERGIC/Golgi vesicles and binds a self-antigen on intracellular membranes via its Fab region and simultaneously the FcμR via its Fc portion. This *cis* engagement may prevent further differentiation of such autoreactive B cells, thereby contributing to the peripheral tolerance to self-antigens located on intracellular membranes (Figure 4). Based on this hypothesis, Mott cell IgMs in *Fcmr* KO mice are anticipated to have autoantibody activity to intracellular membrane components.

Instead of IgM-opsonized self-antigens, it may be equally possible that DNA and DNA-associated autoantigens or RNA and RNA-associated autoantigens are recognized by the respective IgM on BM immature B cells and delivered to an endosomal or lysosomal compartment where TLR9 or TLR7 binds the corresponding ligand-containing IgM BCR. The resultant oligomerized IgM BCRs are transported via a retrograde route to the TGN where FcμR may bind the Cμ3/Cμ4 domain of the oligomeric IgM BCR. In summary, based on the findings of enhanced autoantibody production in all *Fcmr* KO mice and Mott cell formation in our mutant mice as well as the *cis* engagement of FcμR, we propose a model for how FcμR on B cells plays a regulatory role in central and peripheral tolerance.

## FcμR in Diseases

The association of FcμR with human CLL has long been suggested, dating back to studies showing that CLL B cells could form rosettes with ox erythrocytes coated with IgM antibody (84, 85). By flow cytometric assays CLL B cells also exhibited specific IgM binding (57, 86). Subsequently, several investigators showed enhanced *TOSO/FCMR* gene expression in CLL and initially considered that this enhancement would contribute to increased resistance of CLL cells to apoptosis (87, 88). We also examined the surface expression of FcμR by B and T cells in CLL using receptor specific mAbs by flow cytometry. CLL B cells (CD19^**+**^/CD5^**+**^) expressed significantly much higher levels of surface FcμR than B cells from healthy donors. This enhancement was more evident in Ig HC variable region (*IGHV*)-mutated, better prognostic, CD38^**−**^ or early Rai-stage CLL than in *IGHV-*unmutated, poor prognostic, CD38^**+**^ or advanced Rai-stage CLL (89). Intriguingly, surface FcμR levels were also significantly elevated in non-CLL B cells (CD19^**+**^/CD5^**−**^) and T cells (CD19^**−**^/CD5^**+**^), especially in patients with *IGHV-*mutated CLL, when compared with the corresponding populations in healthy individuals. This increase in FcμR expression on T cells in CLL was unique, because normal human T cells activated *ex vivo* with anti-CD3 mAb or PMA down-modulated surface FcμR, whereas normal B cells activated with anti-μ mAb or PMA up-regulated surface FcμR (20). Regarding the enhanced surface expression of FcμR on CLL B cells, CLL-derived BCRs, unlike those from other B cell malignancies, have been shown to ligate each other via interactions between Ig HC CDR3 of one BCR and the framework region 2 of another BCR irrespective of their *IGHV* mutation status, thereby providing antigen-independent cell-autonomous signaling (90, 91). This antigen-independent self-ligation of BCR on CLL cells could account for enhanced surface expression of FcμR as well as for the well-known phenomenon of reduced levels of surface IgM and IgD on CLL cells. It remains unclear, however, why surface FcμR levels were also elevated on non-CLL B and T cells in *IGHV-*mutated CLL patients.

Another remarkable finding was the marked elevation of serum titers of FcμR in CLL patients but not in healthy individuals (89). [One exception was an individual who was found 2 years later to have high serum autoantibody titers against dsDNA.] Detection of the serum FcμR was accomplished by sandwich ELISA using two different receptor-specific mAbs. It was resolved as an ~40 kD protein, distinct from the ~60 kD cell surface FcμR and found by proteomic analysis as a soluble form of the receptor (solFcμR), which was encoded by an alternative spliced FcμR transcript resulting from the direct splicing of exon 4 (stalk 2) to exon 6 (CY1), skipping exon 5 (TM). This splicing event resulted in a reading frame shift in exon 6 and generated a novel 70 aa hydrophilic carboxyl tail, thereby confirming the source of the solFcμR. The functional role of solFcμR in CLL and possibly in autoimmune disorders as observed with aforementioned exceptional control individual remains to be elucidated. In this regard it is noteworthy that administration of another form of solFcμR (FcμR EC/IgG Fc) into EAE-susceptible mice ameliorates the disease (44). Collectively, both membrane-bound and soluble forms of FcμR are elevated in patients with CLL.

Since among leukemia/lymphomas CLL uniquely expresses high levels of FcμR on their surface, two types of immunotherapy targeting for the receptor have thus been developed for CLL cells. One is an immunotoxin-coupled IgM Fc (Cμ2-Cμ4) and the other is chimeric antigen receptor-modified T cells using a single chain fragment-containing the variable region of an anti-FcμR mAb (6B10) (92, 93). In both cases, patient CLL B cells appear to be selectively eliminated *in vitro* without affecting the non-leukemic B and T cells. Apart from FcμR in hematologic malignancy, *FCMR-*deficiency has not yet been identified, but based on the data from *Fcmr* KO mice it may belong to hyper-IgM syndrome. Since FcμR is expressed by B, T, and NK cells in humans, the phenotypic abnormalities of *FCMR* deficiency in affected individuals are predicted to be more complex than those in *Fcmr* KO mice. In patients with selective IgM immunodeficiency, we initially predicted that surface FcμR levels might be high because of lack of ligand-induced down-modulation. Contrary to this assumption, cell surface FcμR levels on a particular circulating B cell subset with a MZ phenotype (IgM^+^/IgD^+^/CD27^+^) in such patients were significantly diminished as compared to age-matched controls, but the molecular basis for this reduction remains to be elucidated (94).

In summary, enhanced levels of both the membrane-bound and secretory forms of FcμR are evident in patients with CLL, possibly as the consequence of antigen-independent autonomous self-ligation of BCR on CLL cells.

## Epilogue

It has been known for many years that passive administration of IgM antibody enhances the subsequent antibody responses to antigenic challenge, whereas passive administration of IgG antibody suppresses the response. Complement activation, but not its lytic activity, has so far been implicated as a mechanism for this IgM-mediated enhancement, and the inhibitory FcγR is involved in IgG-mediated suppression (95, 96). The existence of FcμR on a variety of cell types has also been suggested for nearly 50 years by many investigators including us, but the FcμR cDNA was identified just 10 years ago by a functional cloning strategy (20). However, since FcμR turned out to be identical to the Toso cDNA, which was also previously cloned by functional strategy as a potent inhibitor of Fas-mediated apoptosis, there have been lively debates regarding the real function of this receptor, IgM Fc binding vs. Fas-apoptosis inhibition. While we have now a general consensus that this is an authentic FcμR, there have been clear discrepancies in the phenotypic abnormalities reported in five different *Fcmr* KO mice. In this article, we have discussed potential molecular mechanisms underlying some of these discrepancies. One of the remarkable outcomes of our analysis is the finding of restricted H3K27ac and ATAC marks to the 3' *Fcmr* in activated, but not resting, Treg cells and could account for some puzzles in T cell function described in certain *Fcmr* KO mice. Given the fact that all *Fcmr* KO mice are prone to produce autoantibodies accompanied by increased B-1 B cells, we introduce our hypothetical model for how FcμR controls autoantibody production. We see that FcμR has a very important role in immature B cell in the BM to control against the development of autoreactivity in B cell repertoire. We hope that this short article may help to resolve many still existing puzzles and will open new avenues of investigation.

## Author Contributions

PKJ performed the comparative analysis (Table 1). KH analyzed the FcμR ligand binding property (Figure 2) and the phenotype of mutant-O. NO and SS conducted the epigenetic analysis (Figure 3). AR intellectually contributed. HK and FM made the rest of figures (Figures 1, 4) and wrote the paper. All authors listed approved for publication. PKJ was a scholar of the Alexander von Humboldt Foundation.

### Conflict of Interest Statement

The authors declare that the research was conducted in the absence of any commercial or financial relationships that could be construed as a potential conflict of interest.
